# Engineered Ferritin Nanoparticle Vaccines Enable Rapid Screening of Antibody Functionalization to Boost Immune Responses

**DOI:** 10.1002/adhm.202202595

**Published:** 2023-02-24

**Authors:** Mai N. Vu, Emily H. Pilkington, Wen Shi Lee, Hyon‐Xhi Tan, Thomas P. Davis, Nghia P. Truong, Stephen J. Kent, Adam K. Wheatley

**Affiliations:** ^1^ Peter Doherty Institute for Infection and Immunity Department of Microbiology and Immunology The University of Melbourne Melbourne VIC 3010 Australia; ^2^ Australian Research Council Centre of Excellence in Convergent Bio‐Nano Science and Technology Parkville VIC 3052 Australia; ^3^ Monash Institute of Pharmaceutical Sciences Monash University Parkville VIC 3052 Australia; ^4^ Faculty of Pharmaceutics and Pharmaceutical Technology Hanoi University of Pharmacy 10000 Hanoi Vietnam; ^5^ Australia Institute of Bioengineering & Nanotechnology University of Queensland Brisbane QLD 4072 Australia; ^6^ Melbourne Sexual Health Centre and Department of Infectious Diseases Alfred Hospital and Central Clinical School Monash University Melbourne VIC 3004 Australia

**Keywords:** antibody functionalization, COVID‐19 vaccines, ferritin nanoparticles, lymph node targeting, SARS‐CoV‐2

## Abstract

Employing monoclonal antibodies to target vaccine antigens to different immune cells within lymph nodes where adaptive immunity is initiated can provide a mechanism to fine‐tune the magnitude or the quality of immune responses. However, studying the effects of different targeting antibodies head‐to‐head is challenging due to the lack of a feasible method that allows rapid screening of multiple antibodies for their impact on immunogenicity. Here self‐assembling ferritin nanoparticles are prepared that co‐display vaccine antigens and the Fc‐binding domain of *Staphylococcal* protein A, allowing rapid attachment of soluble antibodies to the nanoparticle surface. Using this tunable system, ten antibodies targeting different immune cell subsets are screened, with targeting to Clec9a associated with higher serum antibody titers after immunization. Immune cell targeting using ferritin nanoparticles with anti‐Clec9a antibodies drives concentrated deposition of antigens within germinal centers, boosting germinal center formation and robust antibody responses. However, the capacity to augment humoral immunity is antigen‐dependent, with significant boosting observed for prototypic ovalbumin immunogens but reduced effectiveness with the SARS‐CoV‐2 RBD. This work provides a rapid platform for screening targeting antibodies, which will accelerate mechanistic insights into optimal delivery strategies for nanoparticle‐based vaccines to maximize protective immunity.

## Graphical abstract

The co‐delivery of vaccine antigens and targeting antibodies to different immune cells within lymph nodes can fine‐tune the magnitude of immune responses. Here a rapid nanoparticle vaccine platform is developed to screen multiple targeting antibodies for their immunogenicity. This work provides mechanistic insights into efficient delivery strategies for nanoparticle vaccines to maximize protective immunity.

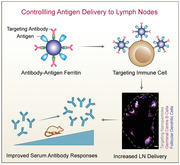

## 1 Introduction

Efficient delivery of vaccine antigens to the lymphoid organs where adaptive immune responses are initiated is critical for successful immunization.^[^ ^1^ ^]^ Attempts to modulate the timing or extent of antigen delivery include the use of depot‐forming adjuvants,^[^ ^2^ ^]^ lipid‐conjugated antigens (e.g., albumin modification),^[^ ^3^ ^]^ or particulate nanocarriers.^[^ ^4^, ^5^, ^6^, ^7^ ^]^ Nanoparticle vaccines can deliver antigens to lymph nodes via passive and active targeting. For example, small‐sized polypropylene nanoparticles (25 nm) are more effectively transported passively via lymphatic capillaries to draining lymph nodes compared to their larger counterparts (≥100 nm).^[^ ^4^ ^]^ Consistently, ferritin nanoparticles delivering influenza^[^ ^7^ ^]^ and HIV antigens^[^ ^8^ ^]^ with a size of approximately 20–40 nm demonstrated increased trafficking and deposition in lymph nodes. Alternatively, nanoparticles can be captured by peripheral dendritic cells (DCs) and actively transported to the lymph node through afferent lymphatics.^[^ ^9^ ^]^ Thus, decorating nanoparticle vaccines with monoclonal antibodies (mAbs) targeting DCs provides a strategy to effectively deliver antigens to the lymph node, potentially improving the resultant immune response.

However, DCs are a heterogeneous cell population comprised of numerous phenotypic subsets with distinct functions in adaptive immune responses.^[^ ^10^ ^]^ For example, mouse CD8*α* ^+^ DCs are mainly responsible for major histocompatibility complex (MHC) I‐cross presentation of antigens to CD8^+^ T cells, priming cytotoxic T cell responses.^[^ ^11^ ^]^ In contrast, CD8*α* ^−^ DCs in mice preferentially process exogenous antigens through the MHC II pathway, promoting CD4^+^ T cell responses and antibody production. Therefore, differential delivery to different DC subsets could alter the relative balance of immune responses generated after immunization. Although cellular immune responses are important for the protection of some vaccines, most vaccines licensed today, including COVID‐19 vaccines, protective efficacy depends primarily on the induction of neutralizing antibodies.^[^ ^12^, ^13^ ^]^ Nanoparticle vaccines functionalized with mAbs that bind to receptors on CD8*α* ^−^ DCs (e.g., CD11b)^[^ ^14^ ^]^ or both CD8*α* ^+^ and CD8*α* ^−^ DCs (e.g., CD11c)^[^ ^15^ ^]^ might increase antigen delivery and presentation to CD4^+^ T cells, driving enhanced antibody immunity via the provision of high‐quality T cell help. Other potential DC targets could be C‐type lectin receptors DEC205 and Clec9a or TNF‐*α* receptor CD40.^[^ ^16^, ^17^, ^18^, ^19^, ^20^ ^]^

Upon reaching lymph nodes, DC‐processed antigens target CD4^+^ T cells for follicular helper T (Tfh) cell induction, while intact unprocessed antigens can be passively transported to subcapsular sinus macrophage (SSM) or follicular dendritic cell (FDC) areas and presented to naïve B cells.^[^ ^21^, ^22^ ^]^ Interactions between antigen‐activated B cells, Tfh cells, and FDCs facilitate the formation of germinal centers (GCs) where B cells undergo proliferation, somatic hypermutation, and selection for antibody clones with increased antigen affinity.^[^ ^23^ ^]^ High‐affinity B cells then differentiate into either memory B cells or antibody‐secreting plasma cells for long‐lived antibody production.^[^ ^6^, ^7^ ^]^ Generation of enhanced GC reactions is a well‐recognized strategy to improve protective antibody responses. Therefore, using antibody‐functionalized nanoparticle vaccines to target receptors on different immune cell subtypes (e.g., CD169^+^ SSMs, FDC‐M1^+^/ CD35^+^/ CD21^+^ FDCs, or CD4^+^ T cells) in the lymph node may provide an alternative pathway to improve vaccine‐induced antibody responses.

Despite the potential of targeting antibody strategy on modulating immunity generated by vaccination, there is currently a lack of a feasible nanoparticle platform that allows rapid assessment of multiple antibodies head‐to‐head for an impact upon immunogenicity. Thus, in this study, we designed ferritin nanoparticles, which self‐assemble from 24 subunits of the ferritin iron‐storage protein^[^ ^7^ ^]^ and have been evaluated as efficient vaccine platforms for various pathogens (e.g., influenza, HIV, or COVID‐19),^[^ ^24^, ^25^, ^26^, ^27^ ^]^ to co‐display both vaccine antigens and a bivalent ZZ (Fc‐binding) domain derived from *Staphylococcus aureus* protein A. This ZZ‐ferritin system allows rapid attachment and screening of antibodies to assess the immunological consequences of targeting during immunization. Using ferritin nanoparticles displaying the model protein antigen – ovalbumin (OVA),^[^ ^4^ ^]^ we screened ten antibodies that target different receptors on subtypes of immune cells, including DCs, SSMs, T cells, and FDCs. We found the highest serum antibody titers induced in mice vaccinated with *α*Clec9a‐functionalized OVA‐ferritin. Increased nanoparticle association with DCs and transport to B cell follicles in draining lymph nodes several hours following vaccination likely contributed to improved GC reactions and subsequent serological antibody outcomes. In contrast, only a modest enhancement was observed after targeting Clec9a receptors with ferritin nanoparticles displaying receptor binding domain (RBD) from SARS‐CoV‐2 spike (S) protein.^[^ ^5^ ^]^ This suggests that the immune boosting effect of this targeting strategy might depend on the nature of both antigen and antibody. This highly tunable targeting system allows rapid immunological screening of antigen and antibody combinations, facilitating rational design of nanoparticle vaccines with optimal delivery strategy for maximal protective immune responses.

## 2 Results

### 2.1 Preparation of Antibody‐Functionalized Ferritin Nanoparticles

**Figure 1:**
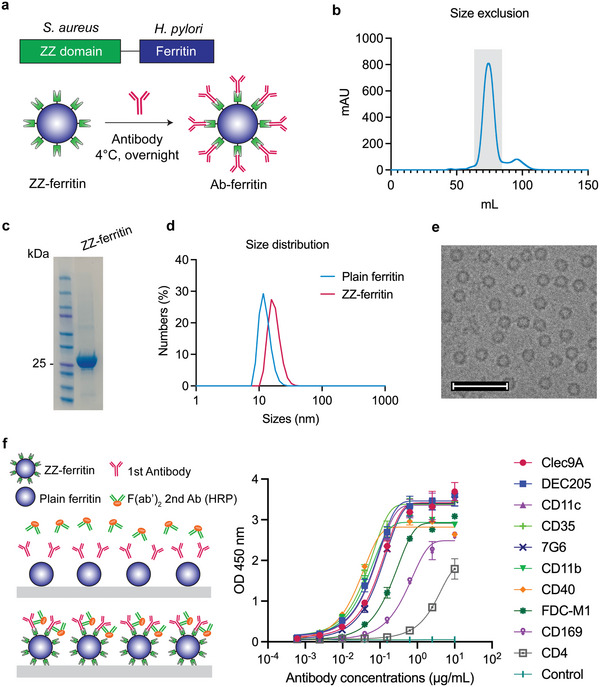
ZZ‐ferritin design and characterization. a) Schematic illustration of ZZ domain of *S. aureus* genetically fused to *H. pylori* ferritin (top) and formation of antibody–ferritin nanoparticle by incubating ZZ‐ferritin and antibody at 4 °C overnight. b) Size exclusion chromatography of ZZ‐ferritin (fraction in the grayed area). c) SDS PAGE showing ZZ‐ferritin with an expected molecular weight of 36 kDa. d) Size distribution of plain ferritin and ZZ‐ferritin measured by DLS. e) Cryo‐EM images of ZZ‐ferritin, scale bars = 50 nm. f) Binding ability of antibodies to ZZ‐ferritin: Schematic illustration of ELISA study design, plain ferritin was used as control (left) and binding affinity of different antibodies with ZZ‐ferritin and plain ferritin control (right).

The Z domain of *Staphylococcus aureus* protein A is a 58‐amino acid protein that specifically binds to CH_2_–CH_3_ regions in the Fc of selected immunoglobulin G (IgG) antibodies.^[^ ^28^ ^]^ We first engineered a dimeric form (ZZ) onto the surface of ferritin nanoparticles (ZZ‐ferritin) to enable facile functionalization with antibodies. The *Helicobacter pylori* ferritin gene, which self‐assembles into a 24‐subunit protein‐based nanoparticle, was genetically fused to the gene encoding the ZZ domain of *Staphylococcal* protein A and expressed in Expi293F cells (**Figure** **1**a). ZZ‐ferritin nanoparticles were purified by ion exchange and size exclusion chromatography (Figure 1b). The correct expression of the ZZ‐ferritin protein with an expected molecular weight of approximately 36 kDa was confirmed by SDS‐PAGE (Figure 1c). Dynamic light scattering (DLS) data indicated an increase in the hydrodynamic diameter of ferritin nanoparticles from ≈12 to ≈20 nm after attaching the ZZ domain to the surface of nanoparticles (Figure 1d), which was further confirmed by cryogenic electronic microscopy (cryo‐EM) (Figure 1e).

We next assessed the binding ability of antibodies to ZZ‐ferritin nanoparticles by ELISA. Briefly, ferritin control or ZZ‐ferritin nanoparticles were coated onto MaxiSorp plates and binding to various antibodies was assessed. F(ab’)_2_ fragment formats of secondary detection antibodies were used to avoid false positive binding with ZZ domains (Figure 1f, left). A library of antibodies tested, their raised species and isotypes, and their main targeted cells are detailed in **Table** **1**. No binding of all antibodies was observed to the plain ferritin control. In contrast, this panel of IgG antibodies showed a range of binding affinities to ZZ‐ferritin nanoparticles (Figure 1f, right and Table 1), with the strength of binding varied depending on species and isotypes. Differences in the amino acid sequence of Fc regions of antibodies raised in different species and isotypes might be responsible for the variety in the binding affinity of antibodies and ZZ‐ferritin.^[^ ^29^ ^]^ Consistent with previous literature,^[^ ^30^ ^]^ human IgG1 and mouse IgG2a antibodies showed the strongest association with ZZ‐ferritin, while lower binding was observed for mouse IgG1 (CD169) and rat IgG2 (FDC‐M1 and CD4).

**Table 1:** Library of antibodies and their characterization: raised species and isotypes, binding affinity to ZZ‐ferritin, and their main targeted cells (+++ strong binding, ++ medium binding, + weak binding)

Antibodies	Species, Isotypes	Binding affinity to ZZ‐ferritin	Main targeted cells
Clec9a	Hu IgG1, *κ*	+++	Dendritic cells
DEC205	Hu IgG1, *κ*	+++	Dendritic cells
CD11c	Hu IgG1, *κ*	+++	Dendritic cells
CD35	Hu IgG1, *κ*	+++	Follicular dendritic cells
7G6	Hu IgG1, *κ*	+++	Follicular dendritic cells
CD11b	Ms IgG2a, *κ*	+++	Dendritic cells
CD40	Ms IgG2a, *κ*	+++	Dendritic cells
FDC‐M1	Rat IgG2c. *κ*	++	Follicular dendritic cells
CD169	Ms IgG1, *κ*	++	SCS macrophages
CD4	Rat IgG2b, *κ*	+	T cells

### 2.2 Antibody Functionalization of OVA Ferritin Vaccine Can Drive Increased Antibody Responses in Mice

**Figure 2:**
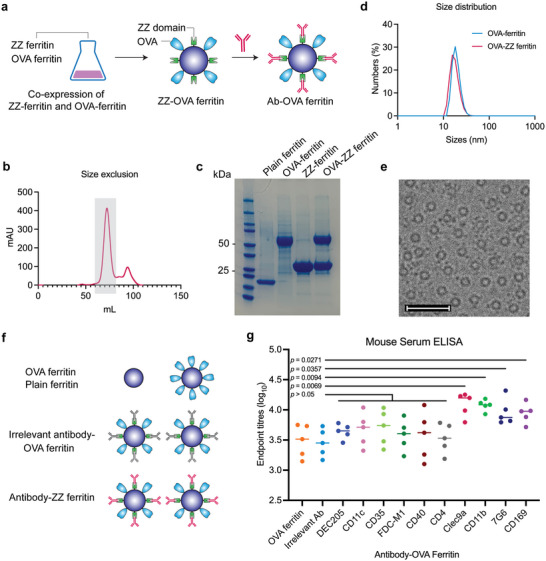
Antibody‐OVA ferritin preparation, characterization, and mice immunization. a, Schematic illustration of OVA‐ZZ formation by co‐expression of OVA‐ferritin and ZZ‐ferritin genes, and formation of Ab‐ZZ ferritin by incubating OVA‐ZZ ferritin with desired antibodies. b, Size exclusion chromatography of OVA‐ZZ ferritin (fraction in the grayed area). c) SDS PAGE showing two bands of OVA‐ZZ ferritin corresponding to OVA‐ferritin and ZZ‐ferritin. d) Size distribution of OVA‐ferritin and OVA‐ZZ ferritin measured by DLS. e) Cryo‐EM images of OVA‐ZZ ferritin nanoparticles, scale bars = 50 nm. f) Experimental design of the serological study: Groups of C57BL/6 mice (*n* = 5) were intramuscularly immunized with either mixture of plain ferritin and OVA‐ferritin, irrelevant antibody‐OVA ferritin, or tested Ab‐OVA ferritin at an equivalent amount of 3.5 µg OVA antigen and 3.0 µg ferritin. g) OVA‐specific IgG titers measured in mouse sera at day 14 post vaccination using an ELISA assay. Each dot represents one mouse and data are representative of two independent experiments. Statistical significance was determined by one‐way ANOVA with Tukey's pairwise comparisons post‐hoc test.

We next modified ferritin nanoparticles to co‐display prototypic vaccine antigens with targeting antibodies on a single particle. We co‐transfected a plasmid encoding ZZ‐ferritin with a plasmid expressing OVA‐ferritin at an equal mass ratio, which upon expression yielded ferritin nanoparticles that co‐displayed both OVA and ZZ domains (**Figure** **2**a).^[^ ^31^ ^]^ OVA‐ZZ ferritin was purified by ion exchange and size exclusion chromatography as before (Figure 2b), with SDS‐PAGE showing two bands of OVA‐ZZ ferritin corresponding to OVA‐ferritin and ZZ‐ferritin at their expected molecular weights (Figure 2c). The chimeric OVA‐ZZ ferritin nanoparticles had a hydrodynamic diameter of ≈20 nm, which was comparable to the size of OVA‐ferritin nanoparticles (Figure 2d). Cryo‐EM images confirmed the successful formation of spherical ferritin nanoparticles at the expected size range (Figure 2e).

To form ferritin nanoparticles that co‐displayed targeting antibodies and OVA antigens, antibodies were incubated with OVA‐ZZ ferritin at a ratio of 1:10 at 4 °C overnight. The effect of targeting antibodies on vaccine immunogenicity was next assessed. Female C57BL/6 mice (*n* = 5 per group) were intramuscularly immunized into hind quadriceps with either targeting antibody–OVA ferritin, irrelevant anti‐human antibody–OVA ferritin, or a mixture of plain ferritin and OVA‐ferritin with each group receiving an equivalent amount of 3.5 µg OVA and 3.0 µg ferritin (Figure 2f, left). Addavax (a squalene‐based oil‐in‐water emulsion) at 50% of the total injection volume was used as an adjuvant for all groups.^[^ ^7^ ^]^ On day 14 after vaccination, we found no significant increases in serum OVA‐specific IgG titers of mice immunized with OVA‐ZZ ferritin functionalized with anti‐DEC205, CD11c, CD35, FDC‐M1, CD40, and CD4 antibodies compared to two untargeted control groups (*p* > 0.05, Figure 2g). In contrast, nanoparticles functionalized with anti‐Clec9a, CD11b, 7G6, and CD169 antibodies elicited significant increases ranging from ≈2.6‐ to ≈4.1‐fold in OVA‐specific IgG levels compared to controls (*p* < 0.05). This demonstrated that antibody targeting was a pathway to potentially modulate the immunogenicity of protein vaccine. Anti‐Clec9a, CD11b, 7G6, and CD169 antibodies were selected for subsequent in‐depth immunogenicity studies.

### 2.3 Antibody‐Functionalized OVA Ferritin Nanoparticles Enhance Antigen‐Specific Germinal Center Responses

**Figure 3:**
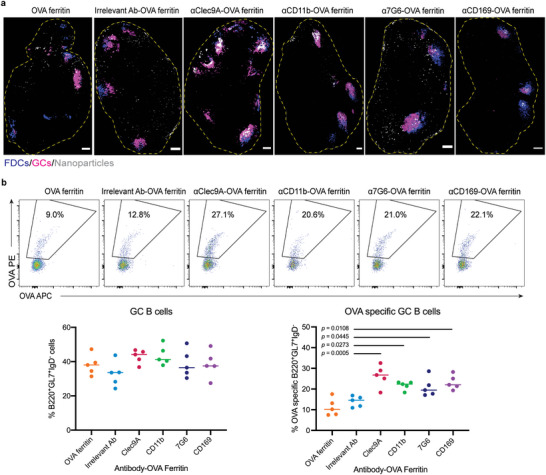
Germinal center responses in lymph nodes of mice immunized with antibody–OVA ferritin. Six groups of C57BL/6 mice (*n* = 5) were intramuscularly immunized with either mixture of plain ferritin and OVA‐ferritin, irrelevant antibody‐OVA ferritin, or tested antibody–OVA ferritin at an equivalent amount of 3.5 µg OVA antigen and 3.0 µg ferritin. At day 14 post‐immunization, inguinal and iliac lymph nodes were harvested and processed. a) Deposition of antibody‐OVA ferritin in FDC and GC areas of draining lymph nodes. Distribution of ferritin nanoparticle fluorescently labeled with AF647 (gray) in whole lymph nodes (in yellow boundaries) stained with CD35 BV421 (FDC—blue) and GL7 AF488 (GCs—magenta). Images were captured by a Zeiss LSM780 inverted confocal microscope. Scale bars = 150 µm. b) Representative flow plot of OVA‐specific GC B cell populations (IgD^−^B220^+^GL7^+^CD38^lo^) (top) and frequencies of GC B cells in total B220^+^IgD^−^ B cells (bottom, left) and OVA‐specific GC B cells in total GC B cells (bottom, right). Each dot represents one mouse and data are representative of two independent experiments. Statistical significance was determined by one‐way ANOVA with Tukey's pairwise comparisons post‐hoc test.

Lymph node germinal centers (GC) are sites where vaccine antigens are deposited upon FDCs and drive the competition of antigen‐specific B cell clones for the eventual production of high‐affinity serum antibodies. To examine the effect of antibody targeting upon GC formation and the trafficking of vaccine antigens, we performed histological studies to visualize the distribution of the nanoparticle vaccines within lymph nodes of mice immunized with either *α*Clec9a‐, *α*CD11b‐, *α*7G6‐, and *α*CD169‐OVA ferritin nanoparticles or two untargeted control groups. On day 14 after immunization, sections of inguinal lymph nodes draining the site of vaccination (quadricep muscle) were labeled with CD35 BV421 and GL7 AF488 for the staining of FDC and GC B cells, respectively. Nanoparticles were visualized based upon pre‐immunization conjugation to Alexa Fluor 647 (AF647). Concentrated depositions of all four antibody–OVA ferritin nanoparticles within CD35^+^ FDC and GL7^+^ GC B cell areas in lymph nodes were observed (**Figure** **3**a). This focused deposition was not evident in animals immunized with untargeted nanoparticles, suggesting antibody targeting could increase the deposition of antigen to GCs of draining lymph nodes in vaccinated mice.

We next evaluated the magnitude and specificity of GC responses using flow cytometry and recombinant OVA probes.^[^ ^6^ ^]^ Mice were immunized as before and single‐cell suspensions were collected from the draining inguinal and iliac lymph nodes on day 14 after vaccination. The gating of total GC B cells (IgD^−^B220^+^GL7^+^CD38^lo^) and OVA‐specific GC B cells are shown in Figures S1a (Supporting Information) and  3b, top respectively. We observed a moderate increase in frequencies of total GC B cells after immunization with *α*Clec9a‐ (median ≈44.2%) and *α*CD11b‐OVA ferritin (median ≈41.3%) when compared to vaccination with two control nanoparticles (median ≈38.1% and ≈33.6%) (Figure 3b, bottom left). In contrast, immunizations with *α*7G6‐ and *α*CD169‐OVA ferritin elicited comparable proportions of GC B cells relative to controls, with medians of ≈36.5% and ≈37.5%, respectively.

In terms of specificity, we found significant increases in frequencies of OVA‐specific GC B cells in mice immunized with the four antibody–OVA ferritin vaccines compared to those vaccinated with untargeted nanoparticles (*p* < 0.05, Figure 3b, bottom right). Notably, *α*Clec9a‐OVA ferritin immunization elicited the highest proportion of OVA‐specific GC B cells, which was approximate twice the frequency seen in control groups (*p* = 0.0005). Overall, these data suggest that focused antigen deposition within the GCs of the draining lymph nodes can drive greater expansion of vaccine‐specific B cells. These effects were most evident targeting Clec9a, CD11b, 7G6, and CD169 receptors, in contrast to *α*DEC205, *α*CD11c, *α*CD35, *α*FDC‐M1, *α*CD40, and *α*CD4 antibodies (Figure S1b, Supporting Information), which did not boost antibody or GC B cell responses.

### 2.4 Anti‐Clec9a‐OVA Ferritin Improves Nanoparticle Delivery to DCs and B Cells in Lymph Nodes

**Figure 4:**
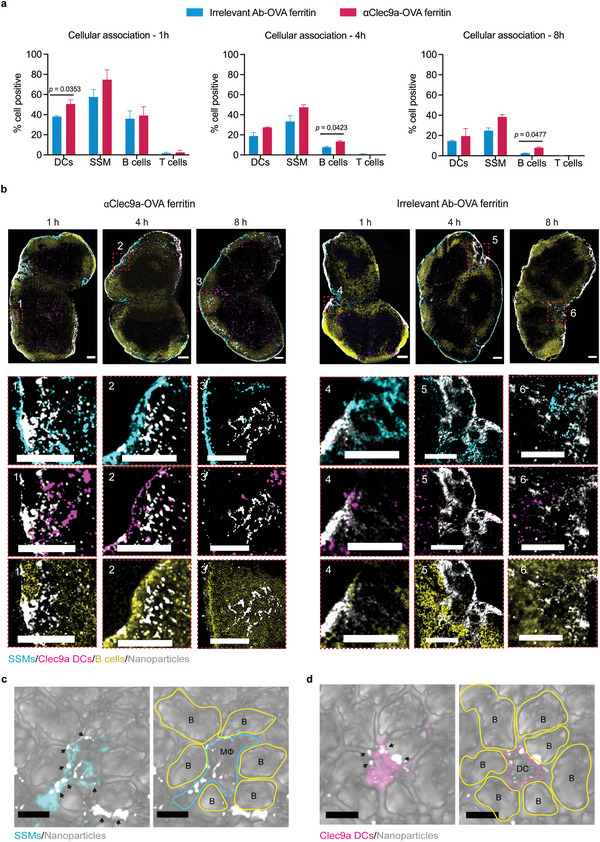
Anti‐Clec9a‐OVA ferritin trafficking and distribution within lymph nodes. Two groups of C57BL/6 mice (*n* = 5) were intramuscularly immunized with αClec9a‐OVA ferritin or irrelevant antibody–OVA ferritin at an equivalent amount of 3.5 µg OVA antigen and 3.0 µg ferritin. a) Percentages of nanoparticle associations with lymph node‐isolated DCs, SSMs, B cells, and T cells at 1 h, 4 h, and 8 h after vaccination. Statistical significance was determined by a paired two‐tailed *t*‐test. b) Distribution of AF647‐labelled ferritin nanoparticles (gray) in lymph nodes stained with CD169 AF488 (SSMs—cyan), Clec9a AF594 (DCs—magenta), and IgD BV421 (B cells—yellow) at 1 h, 4 h, and 8 h after injection. Images were captured by a Zeiss LSM780 inverted confocal microscope. Scale bars = 150 µm. c) SCS macrophage d) Clec9a DC presented nanoparticles (black arrows) to B cells in B‐cell follicles 4 h post‐vaccination with αClec9a–OVA ferritin. Right images are shown as left confocal images with cell boundaries highlighted. Scale bars = 5 µm.

We next assessed nanoparticle trafficking and interactions with immune cells in draining lymph nodes at early time points after immunizations. The anti‐Clec9a antibody, which was stably attached to OVA–ferritin nanoparticles that consistently elicited the highest antibody, was selected for additional trafficking studies (Figures S2 and S3, Supporting Information). We vaccinated mice with Clec9a‐targeted versus untargeted nanoparticles and isolated cells from vaccine‐draining lymph nodes at 1 h, 4 h, and 8 h after injection. The association of nanoparticle vaccines with four major cell types (B cells, T cells, DCs, and SSMs) in the lymph nodes (Figure S4, Supporting Information) was analyzed (**Figure** **4**a). After 1 h, both vaccines reached lymph nodes and showed preferential associations with DCs (≈40–50%), SSMs (≈60–70%), and B cells (≈40%), but minimal binding to T cells (≈2%). While there were negligible differences in percentages of SSMs and B cells associated with these two nanoparticles, the frequency of DC association with Clec9a‐targeted nanoparticles was significantly higher than with untargeted controls (≈1.3‐fold increase, *p* = 0.0353). Interestingly, after 4 and 8 h, there was no marked difference in DC association with the two nanoparticles, while *α*Clec9a‐OVA ferritin displayed a significantly higher proportion of B cell association compared to the controls (≈1.7‐fold, *p* = 0.0423 and ≈3.4‐fold, *p* = 0.0477, respectively). Collectively, these data suggest that within 1 h of injection, both nanoparticle vaccines were effectively trafficked to the lymph nodes, however, targeting with *α*Clec9a could modulate the degree of cellular association.

We performed histological studies to visualize nanoparticle distribution in inguinal lymph nodes after 1 h, 4 h, and 8 h. Lymph nodes were labeled with CD169 AF488, Clec9a AF594, and IgD BV421, markers for SSM, DC, and B cells, respectively, while the vaccines were pre‐labeled with AF647. Consistent with flow cytometry data, after 1 h both *α*Clec9a‐OVA‐ferritin and control nanoparticles were found in lymph nodes and primarily colocalized with macrophages, DCs, and B cells in close proximity to the subcapsular sinus (Figure 4b and Figure S5, Supporting Information). *α*Clec9a‐OVA ferritin was able to translocate from the SCS (1 h) first to the edge (4 h), and then to the center of B cell follicles (8 h). The nanoparticles appeared to co‐localize with SSMs, Clec9a DCs, and B cells (Figure 4c,d). In contrast, only a small proportion of untargeted nanoparticles were detected inside the B cell follicles and remained mainly distributed in the sinus, medulla, or B cell zone border where they appeared to interact with SSMs and/or DCs. Overall, these data suggest that while both vaccines efficiently traveled to lymph nodes, *α*Clec9a‐OVA ferritin nanoparticles could target DCs in vivo and were more likely to be transported to B cell follicles.

### 2.5 Anti‐Clec9a‐RBD Ferritin Nanoparticles Induce Modest Immunogenicity Enhancement

**Figure 5:**
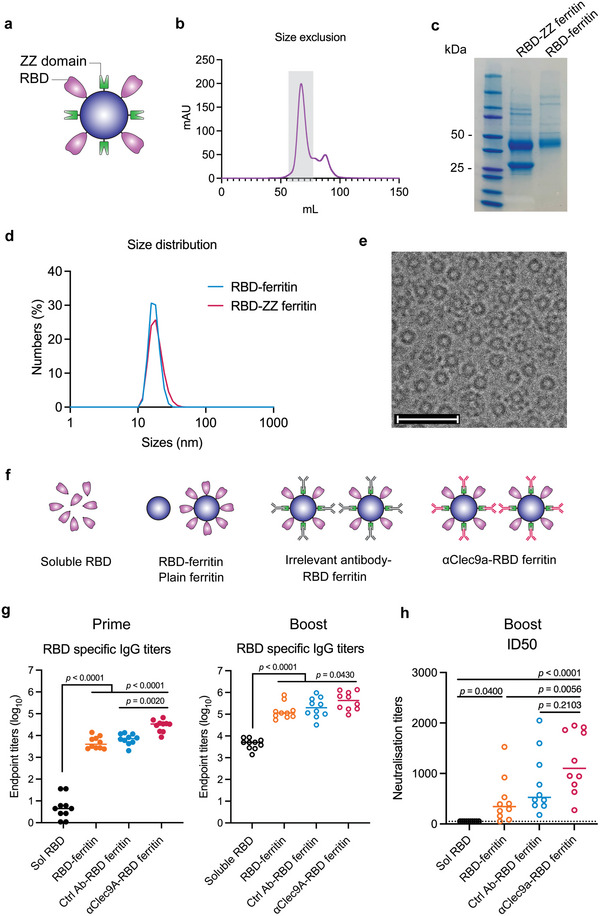
Anti‐Clec9a‐RBD ferritin induces minimal immunogenicity enhancement. a) Schematic illustration of ferritin nanoparticles that co‐display of RBD and ZZ domains. b) Size exclusion chromatography of RBD‐ZZ ferritin (fraction in grayed area). c) SDS PAGE showing two bands of RBD‐ZZ ferritin corresponding to RBD‐ferritin and ZZ‐ferritin. d) Size distribution of RBD‐ferritin and RBD‐ZZ ferritin measured by DLS. e) Cryo‐EM images of RBD‐ZZ ferritin nanoparticles, scale bars = 50 nm. f, Experimental design of serological study: Groups of C57BL/6 mice (*n* = 5) were primed and boosted (at day 21 post prime) with either soluble RBD, mixture of plain ferritin and RBD‐ferritin, irrelevant antibody–RBD ferritin, or αClec9a‐RBD ferritin at an equivalent amount of 4.2 µg RBD antigen and 3.0 µg ferritin. g) RBD‐specific IgG titers measured in mouse sera at day 21 post prime (left) and day 14 post boost (right) using an ELISA assay. h) Serum neutralization titers assessed at day 14 post boost using a micro‐neutralization assay. Each dot represents one mouse and data are from two independent experiments. Statistical significance was determined by a one‐way ANOVA with Tukey's pairwise comparisons post‐hoc test.

We next examined the impact of antibody targeting using a ferritin nanoparticle displaying a ZZ domain and the RBD of SARS‐CoV‐2 spike. RBD contains critical epitopes for the induction of neutralizing antibody responses against SARS‐CoV‐2, but is relatively poorly immunogenic compared to trimeric whole spike immunogens.^[^ ^32^ ^]^ Therefore, boosting the immunogenicity of RBD could be an important strategy for next‐generation SARS‐CoV‐2 vaccine strategies. RBD‐ZZ ferritin nanoparticles were prepared and purified as before (**Figure** **5**a,b), with two bands corresponding to RBD‐ferritin and ZZ‐ferritin proteins seen at expected molecular weights (Figure 5c). Hydrodynamic diameters of RBD‐ and RBD‐ZZ ferritin nanoparticles were of approximately 20 nm (Figure 5d), which were further confirmed by cryo‐EM images (Figure 5e). Anti‐Clec9a antibody was next incubated with the RBD‐ZZ ferritin at a mass ratio of 1 to 10 at 4 °C overnight to form *α*Clec9a‐RBD ferritin nanoparticles, which were shown to be stable after 14 d in the storage conditions (Figure S6, Supporting Information).

Based on our OVA data, we hypothesized that *α*Clec9a‐RBD ferritin nanoparticles would enhance serum antibody responses relative to soluble RBD and untargeted RBD–ferritin in vaccinated mice. C57BL/6 female mice (*n* = 5 mice per group) were intramuscularly vaccinated into hind quadriceps with either *α*Clec9a‐RBD ferritin, irrelevant antibody–RBD ferritin, a mixture of plain ferritin and RBD‐ferritin, or soluble RBD at an equivalent amount of 4.2 µg RBD antigen and 3.0 µg ferritin (Figure 5f). On day 21 after prime, we found that displaying RBD on nanoparticles significantly increased RBD‐specific IgG titers in vaccinated animals compared to those immunized with soluble RBDs (*p* < 0.0001) (Figure 5g). We also observed additional benefit from antibody targeting that *α*Clec9a‐ferritin vaccination induced consistent improvement in anti‐RBD serum antibody levels relative to both RBD–ferritin and irrelevant antibody–RBD ferritin immunizations (≈6.0‐fold, *p* < 0.0001 and 4.1‐fold, *p* = 0.0020, respectively).

Primed mice were boosted at 3 weeks with the same vaccines as their first dose, and RBD‐specific antibody titers on day 14 post‐booster were evaluated. We observed significant increases in serum RBD antibody titers after boosting in all four vaccine groups. While *α*Clec9a‐RBD ferritin vaccination still elicited the highest serum titers of RBD antibody, improvements relative to the irrelevant‐RBD ferritin control were minor and failed to reach statistical significance (≈2.0‐fold increase, *p* > 0.05). We next measured serum neutralization activity of vaccinated animals on day 14 post boost using a live SARS‐CoV‐2 neutralization assay (Figure 5h). While significant improvements in neutralization activity were observed in the serum of mice immunized with *α*Clec9a‐RBD ferritin compared to soluble RBD (≈24.7‐fold, *p* < 0.0001) and RBD‐ferritin (≈2.7‐fold, *p* = 0.0056), there was only minimal enhancement compared to the untargeted nanoparticle control (≈1.5‐fold, *p* = 0.2103).

We next examined the localization of antigens and biogenesis of B cell/GC responses in the lymph nodes of vaccinated mice as before. Focused deposition of RBD‐ferritin was observed in areas enriched with GL7^+^ GC and CD35^+^ FDC irrespective of the use of targeting antibodies and in sharp contrast to antigen distribution observed after soluble RBD vaccination (Figure S7a, Supporting Information). The magnitude and specificity of GC responses were assessed using flow cytometry with RBD and spike probes.^[^ ^32^ ^]^ Irrespective of the co‐display of targeting antibodies, RBD‐ferritin nanoparticles elicited significantly higher frequencies of both total GC B cells (*p* < 0.0001) and RBD‐specific GC B cells (S^+^RBD^+^ GC B cells, *p* = 0.0001) than in animals immunized with soluble RBD (Figure S7b, Supporting Information). Consistent with antibody titers, *α*Clec9a‐RBD ferritin vaccination increased the frequency of total and RBD‐specific GC B cells (*p* = 0.0111 and *p* = 0.0063, respectively) relative to RBD‐ferritin immunizations. However, the improvements were minor when compared to irrelevant antibody–RBD ferritin and did not reach statistical significance (*p* = 0.3095 and *p* = 0.1802, respectively). Overall, our data suggest that displaying RBD on nanoparticle scaffolds significantly augmented the immunogenicity and neutralization activity elicited by SARS‐CoV‐2 RBD immunogens, with any additional benefit of targeting to Clec9a leading to comparatively minor increases in the titers of anti‐RBD serum antibodies.

## 3 Discussion

Trafficking and distribution of vaccine antigens within secondary lymphoid organs are critical steps in the development of effective adaptive immune responses. Nanoparticle vaccines provide a tunable platform to enable antibody‐based targeting of vaccine immunogens to different subtypes of immune cells in vivo, providing a pathway for potential modulation of immunity generated by immunization. We utilized ZZ domains to form non‐covalent bindings with antibodies, which offers several advantages over commonly used covalent conjugation techniques, monomeric Z, or full‐length protein A. First, ZZ domains allowed rapid functionalization without risking the chemical degradation of the vaccine or antibody. For example, cysteine covalent coupling with maleimide requires the use of a reducing reagent to create free reactive groups,^[^ ^33^ ^]^ which may alter or destroy important linkages (e.g., disulfide bonds).^24^ Second, by binding to the Fc fragment of IgG, ZZ domains correctly orient antibodies on the nanoparticle surface to maximize targeting potential. Third, bivalent ZZ domains were shown to bind more strongly to Fc than monomeric Z domain, while maintaining a smaller molecular weight (≈16 kDa) compared to full‐length protein A (≈70 kDa), minimizing changes to overall size and impact on the expression or self‐assembly of ferritin nanoparticles.

Ferritin nanoparticles have been employed to deliver vaccine antigens for various models of infectious diseases such as influenza, HIV, or COVID‐19.^[^ ^24^, ^25^, ^26^ ^]^ In these studies, antigen‐carried ferritin nanoparticles were demonstrated to elicit enhanced GC responses and superior immunogenicity relative to soluble antigens. However, here we established a system to use ferritin to co‐deliver both vaccine antigens and targeting antibodies on a single particle, in order to direct antigens to specific immune cell types in an attempt to improve humoral immunity. This modular system allows for the rapid evaluation of commercially sourced targeting antibodies derived from a diverse range of species or isotypes. We observed increased antibody responses after vaccinations with four antibodies when functionalized on OVA‐ferritin nanoparticles, including *α*Clec9a, *α*CD11b, *α*7G6, and *α*CD169, which target Clec9a^+^CD8*α* ^+^ DCs, CD11b^+^CD8*α* ^−^ DCs, CD35^+^/CD21^+^ FDCs, and CD169^+^ SSMs, respectively. With the exception of Clec9a^+^CD8*α* ^+^ DCs, these other cells are all directly involved in the antibody production pathway, either by activating CD4^+^ T cells or B cells in the lymph node, and thus promoting GC formation.^[^ ^34^ ^]^ Augmented GC B cell activity was previously confirmed to positively correlate with improved serum antibody outcomes.^[^ ^6^, ^23^, ^28^, ^35^ ^]^ On the other hand, although CD8*α* ^+^ DCs are mainly responsible for priming cytotoxic CD8^+^ T cells, previous studies suggested that targeting antigens to Clec9a^+^ DCs generated potent Tfh and/or GC B cell responses, and therefore, strong antibody responses in mice and non‐human primates.^[^ ^18^, ^19^, ^36^, ^37^ ^]^ Similarly, in this study, we also observed increased frequencies of antigen‐specific GC B cells and robust serum antibody titers in mice vaccinated with *α*Clec9a–OVA ferritin nanoparticles.

Two potential mechanisms that might contribute to improved GC responses after *α*Clec9a–OVA ferritin vaccination in mice were explored: i) increased nanoparticle trafficking to lymph nodes and association with DCs, and ii) improved antigen delivery to B cell follicles. Entry of vaccine antigens to lymph nodes can occur by both passive and active transport pathways. Vaccine antigens and nanoparticles with a size of <50 nm can accumulate efficiently in lymph nodes after passive transport via the lymphatics.^[^ ^4^, ^38^, ^39^, ^40^ ^]^ OVA ferritin nanoparticles, with a hydrodynamic size of ≈20 nm would be expected to effectively drain directly to the lymph node. In fact, both *α*Clec9a‐ and untargeted OVA–ferritin nanoparticles accumulated in draining lymph nodes 1 h after vaccination. While both nanoparticles were associated with DCs, SSMs and B cells, *α*Clec9a‐OVA ferritin showed a greater association with DCs than OVA–ferritin controls, suggesting active targeting might contribute to the greater density of *α*Clec9a–OVA ferritin nanoparticles in lymph nodes after immunization. At later time points, targeting of Clec9a appeared to drive improved delivery of *α*Clec9a–OVA ferritin nanoparticles to B cell follicles compared to untargeted controls. We also observed *α*Clec9a–OVA ferritin nanoparticles in contact with B cells at the edge of B cell zones, consistent with a study suggesting Clec9a DCs might directly present antigens to B cells, primarily at the B cell border.^[^ ^37^ ^]^ Irrespective of the precise delivery modality, the increased delivery of vaccine antigens to the lymph node by *α*Clec9a–OVA ferritin appeared to drive enhanced GC reactions and serum antibody responses.

A ZZ‐ferritin system co‐displaying SARS‐CoV‐2 RBD confirmed the potential utility of nanoparticles coupled with targeting to improve immune responses to RBD immunogens. Our group and others previously reported relatively poor immunogenicity of soluble RBD immunogens, which was associated with limited GC activity.^[^ ^32^, ^41^, ^42^ ^]^ The arrangement of multiple RBDs on a particulate platform including ferritin nanoparticles was demonstrated to elicit more robust and persistent neutralizing antibody responses in mice and nonhuman primates.^[^ ^26^, ^41^, ^42^, ^43^, ^44^ ^]^ Similarly, in this study, we observed enhanced serum antibody titers and GC reactions after both priming and boosting mice with RBD‐ferritin nanoparticles in comparison with soluble RBD. While the incorporation of *α*Clec9a antibody on RBD ferritin did increase antibody titers and serological neutralizing activity against SARS‐CoV‐2 compared to RBD ferritin alone, any improvements were relatively modest. This suggests that factors such as intrinsic immunogenicity and/or biochemical nature of a given vaccine immunogen may impact the overall immune benefits of in vivo targeting.

## 4 Conclusion

Overall, we have demonstrated a modular nanoparticle vaccine platform that enables the co‐delivery of vaccine antigens while allowing rapid screening of targeting antibodies. Targeting different immune cell types in vivo was shown to modulate vaccine immunogenicity, potentially by increasing the density of vaccine antigens in relevant areas of the draining lymph node. Although this work only focussed on augmenting humoral immunity which is critical in protection from SARS‐CoV‐2, influenza, and other acute respiratory diseases,^[^ ^12^, ^13^ ^]^ future studies using this facile vaccine model could address targeting requirements for enhanced cellular immunity, which may be more important with chronic infections such as *Mycobacterium tuberculosis*.^[^ ^45^ ^]^ A greater understanding of the optimal dynamics of antigen delivery to maximize vaccine immunogenicity and/or efficacy will inform further advances in vaccine nanotechnology.

## 5 Experimental Section

### Expression of ZZ‐Ferritin, OVA‐Ferritin, OVA‐ZZ Ferritin, RBD‐Ferritin, and RBD‐ZZ Ferritin Nanoparticles

To produce ZZ‐ferritin, OVA‐ferritin, and RBD‐ferritin, a gene encoding the ZZ domain of *Staphylococcal* protein A, OVA, or RBD respectively was fused to a modified gene encoding *H. pylori* ferritin, which was cloned into a mammalian expression vector. DNAs were purified using Plasmid Maxi Kit (QIAGEN) and transfected into Expi293F cells (Life Technologies) using ExpiFectamine 293 transfection reagents. To form OVA‐ZZ ferritin or RBD‐ZZ ferritin, a 1:1 mass ratio of plasmids encoding ZZ‐ferritin and OVA/RBD‐ferritin were co‐expressed in the Expi293F cells. The ferritin nanoparticles were then purified using ion exchange chromatography with the HiTrap Q HP column (GE Healthcare), followed by size exclusion chromatography using the Superose 6 column (GE Healthcare). The correct expression of ZZ‐ferritin, OVA–ferritin, RBD‐ferritin, OVA–ZZ ferritin, and RBD‐ZZ ferritin were then confirmed by SDS‐PAGE. For imaging studies, ferritin nanoparticles were fluorescently labeled with Alexa Fluor 647 (AF647) using AF647 protein labeling kits (Life Technologies).

### Characterization of Ferritin Nanoparticles:

Dynamic light scattering (DLS): A solution of 50 µg mL^−1^ nanoparticles in PBS was used to measure the dynamic diameter of the ferritin nanoparticles using a Malvern Zetasizer Nano Series with a 4 mW He–Ne ion laser (*λ* = 633 nm). The measurement was carried out three times at 25 °C.

Fluorescence measurement: Fluorescence spectra of ferritin particles were obtained using a fluorescence spectrophotometer (Shimadzu RF‐501 PC) with an excitation of 635 nm and slit widths of 5 nm for both excitation and emission. Cryogenic Electron Microscopy (Cryo‐EM): 4 µL of ferritin nanoparticles in PBS at 2 mg mL^−1^ were placed onto copper grids (200‐mesh) previously coated with holey carbon film (Quantifoil R1.2/1.3) and pre‐glow discharged in a Pelco glow discharge unit. Samples were blotted for 3 s at a blot force of −6 in a Vitrobot plunge freezer system (FEI) and plunged into liquid ethane at 5 °C to form a thin film on the grids. The vitrified samples were held by a Gatan 626 cryoholder and imaged by a Tecnai 12 TEM at a voltage of 120 kV and temperatures at −175 to −170 °C. Images were processed by a Gatan Eagle high‐resolution CCD camera (4000 × 4000).

### Binding of Antibodies to ZZ‐Ferritin Nanoparticles

Direct ELISA was used to assess the binding ability of antibodies to ZZ‐ferritin nanoparticles. A 100 µL ZZ‐ferritin or plain ferritin control solution at 2 µg mL^−1^ in PBS was added to 96‐well MaxiSorp plates (Thermo Fisher Scientific) and incubated overnight at 4 °C. The plates were then blocked with 200 µL of 5% skim milk in PBS for 2h at RT and incubated with a fourfold serial dilution of antibodies of interest with a starting concentration of 10 µg mL^−1^ in 2 h at RT. F(ab’)_2_ secondary antibody conjugated with horseradish‐peroxidase (HRP) (Thermo Fisher) at 1:10000 in 5% skim milk solution was added to the plates and placed for 1 h at RT. The plates were then developed with 80 µL of tetramethylbenzidine (TMB—Sigma Aldrich) in 7 min, and stopped by adding 50 µL of 0.16 m sulfuric acid. Absorbance was measured at 450 nm. The binding ability of antibodies and the ZZ‐ferritin nanoparticles was classified based on the antibody concentration giving signal 3x above background: < 0.01 µg mL^−1^ (strong bindings), <0.1 µg mL^−1^ (medium bindings), >0.1 µg mL^−1^ (weak bindings).

### Antibody Functionalization

To form antibody‐functionalized nanoparticles, purified IgG antibodies were incubated with either ZZ‐ferritin or antigen‐ZZ ferritin nanoparticles at a mass ratio of 1:5 or 1:10 respectively at 4 °C overnight. The association and stability of antibody binding to the ZZ nanoparticles were analyzed by size exclusion chromatography using a Superdex 200 Increase 5/150 GL column (GE Healthcare) with a flow rate of 0.25 mL min^−1^.

### Mouse Immunization

C57BL/6 female mice (6 to 8 weeks old, *n* = 5 per group)^[^ ^6^, ^7^, ^35^ ^]^ were intramuscularly immunized into hind quadriceps with either antibody conjugated antigen‐ZZ ferritin, irrelevant anti‐human antibody–antigen ferritin, or a mixture of plain ferritin and antigen–ferritin as controls at an equivalent amount of 3.5 µg OVA or 4.2 µg RBD along with 3.0 µg ferritin. Addavax at 50% volume was used as an adjuvant for all groups. Mice were injected with 50 µL of vaccine solution in each leg.

### Mouse Serum ELISA

Antigen‐specific IgG titers in vaccinated mice were detected by a direct ELISA. The antigen (either soluble OVA or RBD) at 2 µg mL^−1^ in PBS (100 µL) was added to 96‐well MaxiSorp plates to coat overnight at 4 °C. The plates were then blocked with 200 µL of 5% skim milk in PBS at RT. Mouse serum was diluted in blocking solution, starting from 1:100 ratio with a serial fourfold dilution, which was added to plates and incubated at RT for 2 h. The plates were next placed with F(ab’)_2_ antimouse secondary antibody conjugated with HRP at 1:10000 for 1 h at RT. Developing solution (TMB) was added to the plates for 7 min, followed by halting with 50 µL of 0.16 m sulfuric acid. Absorbance was measured at 450 nm. ELISA endpoint titers were calculated as the reciprocal serum dilution giving signal 3× above background.

### Flow Cytometric Detection of Antigen‐Specific B Cells

Mice were sacrificed, and inguinal and iliac lymph nodes were collected and pooled. Single cells were isolated and stained with Aqua viability dye and Fc‐blocked with anti‐CD16/32 antibody. The cells were then labeled with either OVA‐PE and OVA‐APC probes or spike‐PE and RBD‐APC probes and the following antibodies: F4/80 BV786 (BM8; BioLegend), B220 BUV737 (RA3‐6B2; BD), CD45 Cy7‐APC (30‐F11; BD), IgD BUV 395 (11‐26c.2a; BD), GL7 AF488 (GL7; BioLegend), CD38 Cy7‐PE (clone 90; BioLegend). Cells were then washed twice, fixed in 4% formaldehyde solution, and analyzed on a BD LSR Fortessa using BD FACSDiva. Flow cytometry data were processed using FlowJo v10.

### Microneutralization Assay

SARS‐CoV‐2 isolate CoV/Australia/VIC31/2020 was passaged in Vero cells and stored at −80 °C.^[^ ^46^ ^]^ Infectivity of virus stocks was then determined by titration on HAT‐24 cells (a clone of transduced HEK293T cells stably expressing human ACE2 and TMPRSS2).^[^ ^47^ ^]^ In a 96‐well flat bottom plate, virus stocks were serially diluted five‐fold (1:5–1:78125) in DMEM with 1 µg mL^−1^ TPCK trypsin, added with 60000 freshly trypsinized HAT‐24 cells per well and incubated at 37 °C. After 24 h, 10 µL of alamarBlue Cell Viability Reagent (ThermoFisher) was added into each well and incubated at 37 °C for 1 h. The reaction was then stopped with 1% SDS and read on a FLUOstar Omega plate reader (excitation wavelength 560 nm, emission wavelength 590 nm). The relative fluorescent units (RFU) measured were used to calculate % viability (“sample” ÷ “no virus control” × 100), which was then plotted as a sigmoidal dose–response curve on Graphpad Prism to obtain the virus dilution that induces 50% cell death (50% lethal infectious dose; LD_50_). Virus stocks were titrated in quintuplicate in three independent experiments to obtain mean LD_50_ values.

To determine serum neutralization activity, heat‐inactivated mouse serum samples were diluted 2.5‐fold (1:50–1:30517) in duplicate and incubated with SARS‐CoV‐2 virus at a final concentration of 2 × LD_50_ at 37 °C for 1 h. Next, 60 000 freshly trypsinized HAT‐24 cells in DMEM with 5% FCS were added and incubated at 37 °C. “Cells only” and “Virus+Cells” controls were included to represent 0% and 100% infectivity respectively. After 24 h, 10 µL of alamarBlue Cell Viability Reagent (ThermoFisher) was added into each well and incubated at 37 °C for 1 h. The reaction was then stopped with 1% SDS and read on a FLUOstar Omega plate reader. The relative fluorescent units (RFU) measured were used to calculate % neutralization with the following formula: (“Sample” ‐ “Virus+Cells”) ÷ (“Cells only” ‐ “Virus+Cells”) × 100. IC_50_ values were determined using four‐parameter non‐linear regression in GraphPad Prism with curve fit constrained to have a minimum of 0% and a maximum of 100% neutralization.

### Mouse Immune Cell Association

C57BL/6 female mice (6 to 8 weeks old) were anesthetized by inhalation of isoflurane and intramuscularly injected into hind quadriceps with *α*Clec9a–OVA ferritin or irrelevant antibody–OVA ferritin at an equivalent amount of 3.5 µg OVA antigen and 3.0 µg ferritin. Addavax at 50% of the total volume was used as a vaccine adjuvant for all groups. After 1 h, 4 h, and 8 h, the inguinal and iliac lymph nodes of the injected mice were harvested, pooled, and smashed into single cells. Cell suspensions were placed in 5 mL round bottom polystyrene tubes and stained with Aqua viability dye (Thermo Fisher Scientific) and Fc blocked with anti‐CD16/32 antibody (clone 93, BioLegend). The immune cells were then stained with NK1.1 BUV395 (PK136, BD), B220/CD45R BUV737 (RA3‐6B2, BD), CD35 BV421 (8C12, BD), CD169 BV605 (3D6.112, BioLegend), F4/80 BV650 (BM8, BioLegend), CD11c BV785 (N418, BioLegend), Ly6C FITC (AL‐21, BD), Ly6G PerCP Cy5.5 (1A8, BD), CD3e PE‐CF594 (145‐2C11, BD), CD11b PE‐Cy7 (M1/70, BD) and CD45 APC‐Cy7 (30‐F11, BD). Cells were washed twice, fixed in 4% formaldehyde solution, and analyzed on a BD LSR Fortessa using BD FACSDiva to evaluate nanoparticle associations.

### Confocal Microscopy

C57BL/6 female mice (6 to 8 weeks old) were immunized with AF647‐labeled nanoparticle vaccines as previously described. The lymph nodes of immunized mice were collected in OCT Embedding Compound (Tissue‐Tek) at 1 h, 4 h, and 8 h or 14 or 21 d after vaccination, snap frozen, and stored at ‐80 °C overnight. Tissues were then sectioned into 7 µm slices (Leica) and dried overnight at RT. The tissues were fixed with pre‐chilled acetone (‐20 °C) for 10 min, rehydrated in PBS for another 10 min at RT, and blocked with 5% bovine serum albumin (BSA—Millipore Sigma), and 1:50 rat anti‐mouse CD16/CD32 Fc block. Cells were stained with IgD BV421 (11‐26C.1, BD), Clec9a AF594 (7H11, R&D Systems), and CD169 AF488 (3D6.112, BioLegend) for early time point studies, or with CD35 BV421 (8C12; BD), B220 BV510 (RA3‐6B2, BD), and GL7 AF488 (GL7; BioLegend) for 14‐ or 21 d studies. Slides were washed, mounted with Prolong Diamond Antifade Mountant (Life Technologies), and imaged by Zeiss LSM780 inverted confocal microscopy using Zen Black software. Images were captured with a 20 × 0.8NA air objective at 1 airy unit and a resolution of 512 × 512 pixels with a dwell time of the pixels of 10 µs per pixel.

### Study Approval

Mouse studies were approved by the University of Melbourne Animal Ethics Committee (Ethics numbers: 21704 and 21237).

### Statistical Analysis

Data were presented as mean ± S.D (*n* = 5). Statistical analyses were performed using GraphPad Prism version 9. For analyses comparing two groups, an unpaired two‐tailed *t*‐test was performed. For all other analyses comparing multiple groups, one‐way ANOVA with Tukey's pairwise comparisons post‐hoc test was used. *P* value of less than 0.05 (*p* < 0.05) was considered to be significant for all statistical tests.

## Conflict of Interest

The authors declare no conflict of interest.

## Supporting information

Supporting Information

## Data Availability

The data that support the findings of this study are available from the corresponding author upon reasonable request.
