# Supplementary material for: Engineered Ferritin Nanoparticle Vaccines Enable Rapid Screening of Antibody Functionalization to Boost Immune Responses
Source: Adv Healthc Mater. 2023 Feb 24;12(17):2202595. doi: 10.1002/adhm.202202595 (PMC11469303; doi:10.1002/adhm.202202595)
Supplement: Supplementary file 1 — Supporting Information [file ADHM-12-2202595-s001.pdf]

ADVANCED HEALTHCARE MATERIALS

Supporting Information

for *Adv. Healthcare Mater.*, DOI 10.1002/adhm.202202595

Engineered Ferritin Nanoparticle Vaccines Enable Rapid Screening of Antibody Functionalization to Boost Immune Responses

*Mai N. Vu, Emily H. Pilkington, Wen Shi Lee, Hyon-Xhi Tan, Thomas P. Davis, Nghia P. Truong, Stephen J. Kent and Adam K. Wheatley**

Supporting Information

Engineered Ferritin Nanoparticle Vaccines Enable Rapid Screening of Antibody Functionalisation to Boost Immune Responses

*Mai N. Vu, Emily H. Pilkington, Wen Shi Lee, Hyon-Xhi Tan, Thomas P. Davis, Nghia P. Truong, Stephen J. Kent, and Adam K. Wheatley**

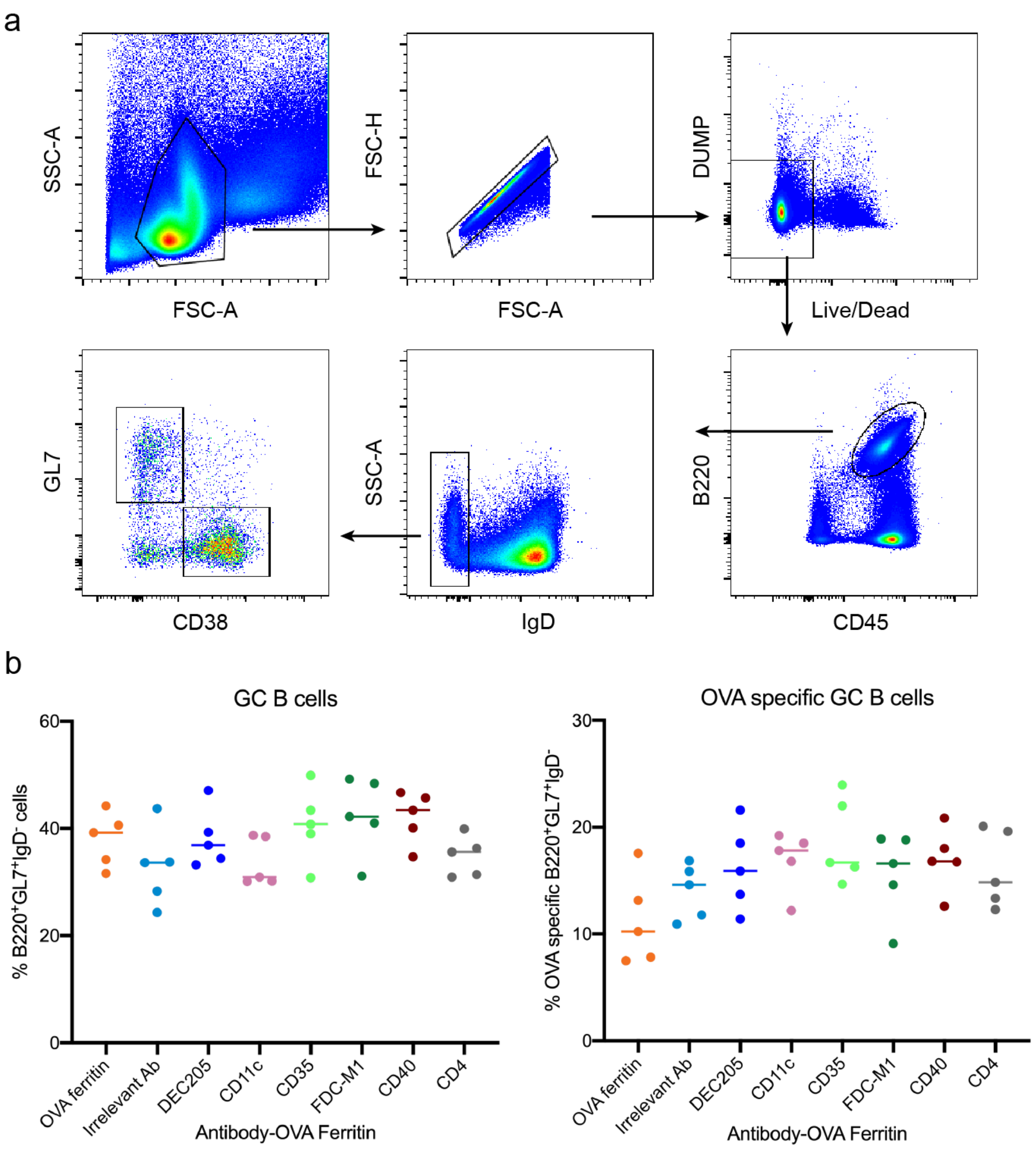

Figure S1: a, Gating strategy to identity GC B cells in mouse lymph nodes. GC B cells are identified as lymphocytes, single cells, Live/Dead$^-$DUMP$^-$, CD45$^+$B220$^+$, IgD$^-$, GL7$^+$CD38^{lo}. **b**, Groups of

C57BL/6 mice (n = 5) were intramuscularly immunised with either mixture of plain ferritin and OVA-ferritin, irrelevant antibody-OVA ferritin, or tested Ab-OVA ferritin at an equivalent amount of 3.5 µg OVA antigen and 3.0 µg ferritin. Frequencies of GC B cells (IgD$^-$B220$^+$GL7$^+$CD38^{lo}) in total IgD$^-$ B220$^+$ B cells (right) and OVA-specific GC B cells in total GC B cells (left). Each dot represents one mouse and data are representative of two independent experiments.

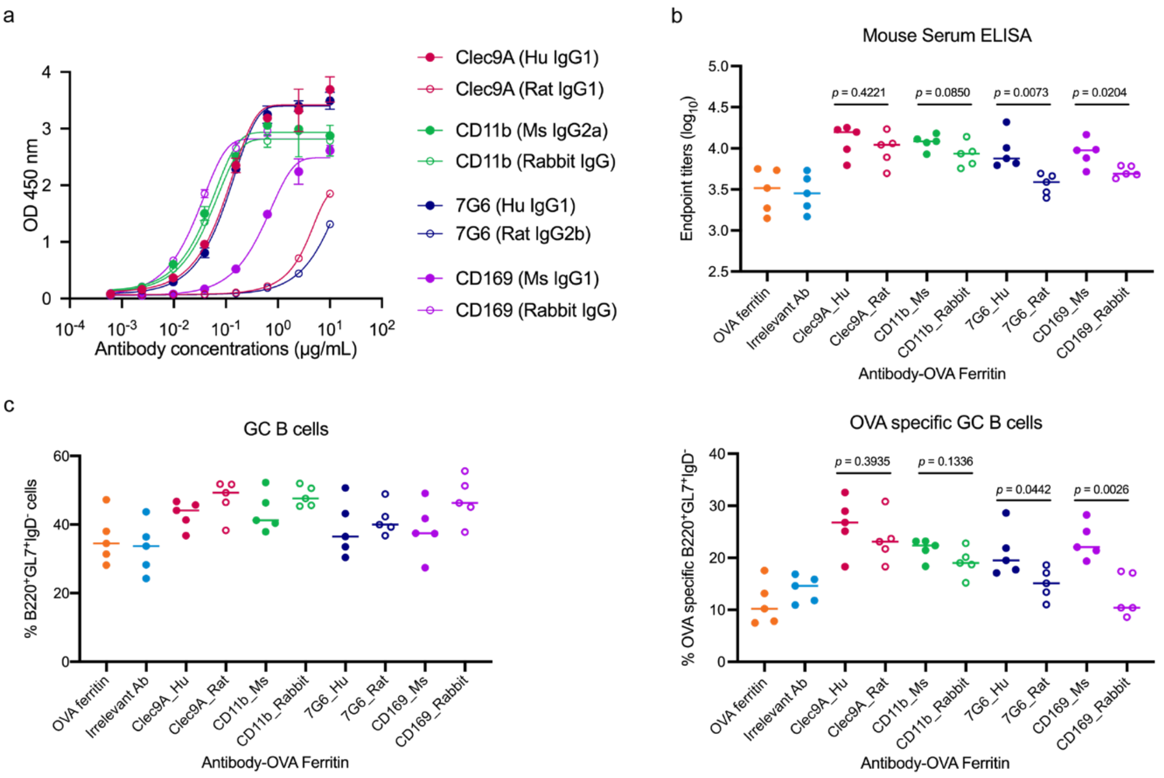

Figure S2: Antibody species affect antibody bindings to ZZ-ferritin and subsequently immune responses in vaccinated mice. a, Binding affinity of four antibodies (αClec9a, αCD11b, α7G6, and αCD169) raised in two different species with ZZ-ferritin assessed by an ELISA assay. **b**, Ten groups of C57BL/6 mice (n = 5) were intramuscularly immunised with either mixture of plain ferritin and OVA-ferritin, irrelevant antibody-OVA ferritin, or tested Ab-OVA ferritin at an equivalent amount of 3.5 µg OVA antigen and 3.0 µg ferritin. OVA-specific IgG titres in mouse sera of vaccinated mice at day 14 post immunisation were calculated using a direct ELISA. **c**, Frequencies of GC B cells (IgD$^-$B220$^+$GL7$^+$CD38^{lo}) in total IgD$^-$B220$^+$ B cells (right) and OVA-specific GC B cells in total GC B cells (left). Each dot represents one mouse and data are representative of two independent experiments. Statistical significance was determined by one-way ANOVA with Tukey's pairwise comparisons post-hoc test.

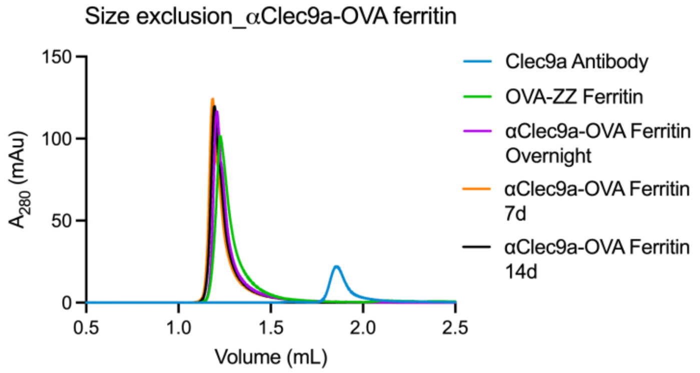

Figure S3: Size exclusion fractions showing the association and stability of Clec9a antibody binding to OVA-ZZ ferritin nanoparticles after overnight incubation and 7 days and 14 days in storage conditions 4 °C.

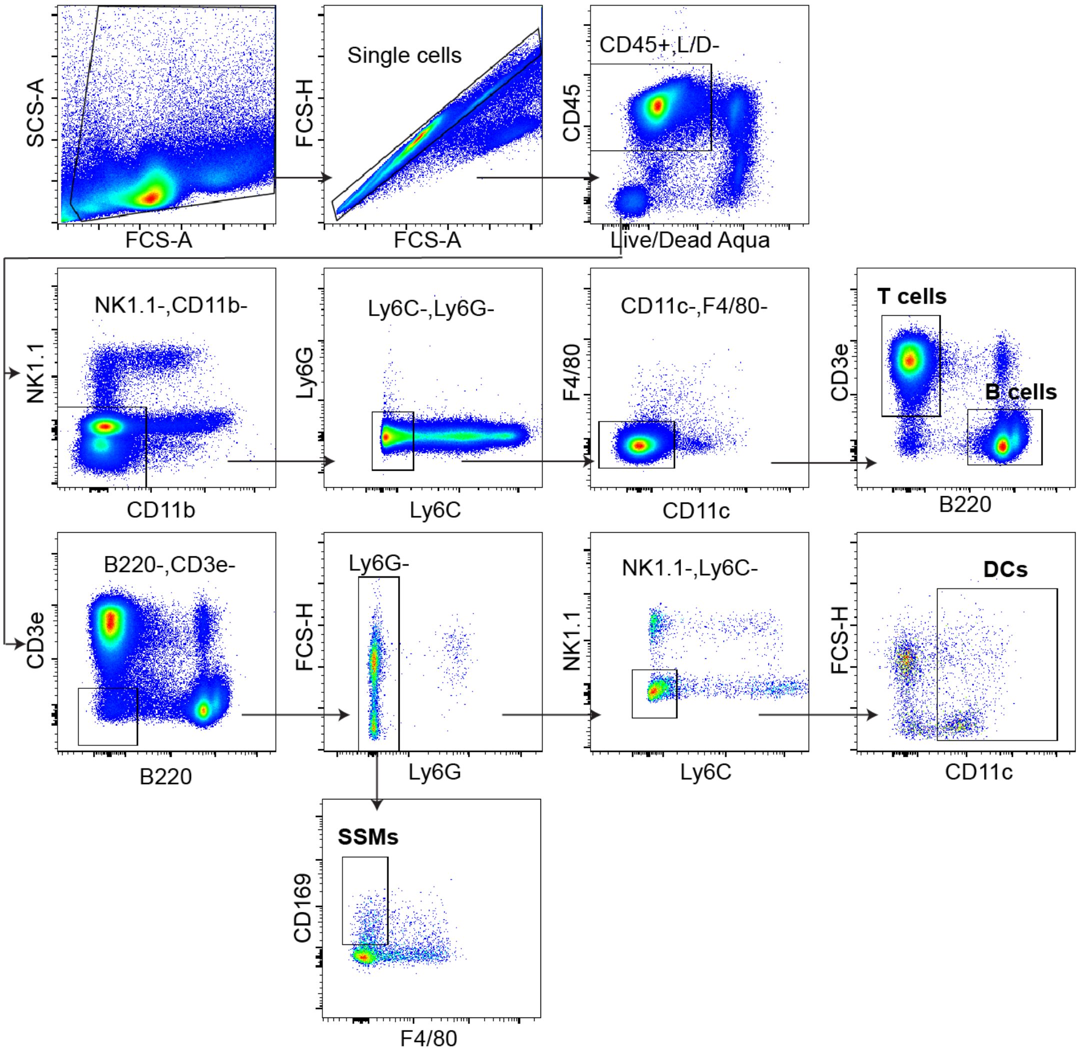

Figure S4: Gating strategy to identity CD169^{+} SSMs, CD11c^{+} DCs, B220^{+} B cells, and CD3^{+} T cells in mouse lymph nodes.

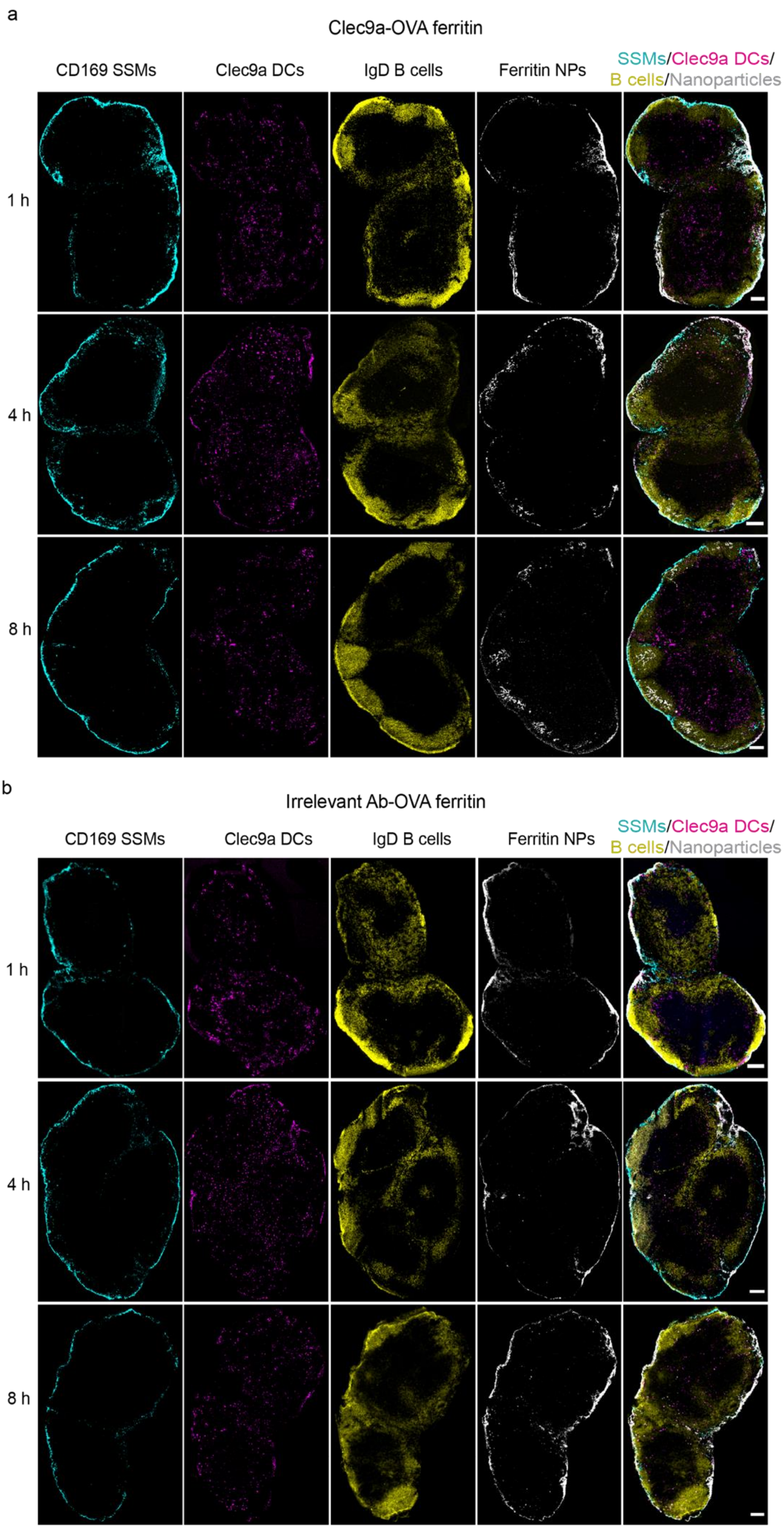
a
Clec9a-OVA ferritin
CD169 SSMs
Clec9a DCs
IgD B cells
Ferritin NPs
SSMs/Clec9a DCs/
B cells/Nanoparticles
1 h
4 h
8 h
b
Irrelevant Ab-OVA ferritin
CD169 SSMs
Clec9a DCs
IgD B cells
Ferritin NPs
SSMs/Clec9a DCs/
B cells/Nanoparticles
1 h
4 h
8 h

Figure S5: Anti-Clec9a-OVA ferritin distribution in lymph nodes. Two groups of C57BL/6 mice (n = 5) were intramuscularly immunised with αClec9a-OVA ferritin or irrelevant antibody-OVA ferritin at an equivalent amount of 3.5 µg OVA antigen and 3.0 µg ferritin. Distribution of **a**, αClec9a-OVA ferritin and **b**, control ferritin nanoparticles (AF647 - gray) in lymph nodes stained with CD169 AF488 (SSMs – cyan), Clec9a (DCs - magenta), and IgD (B cells - yellow) at 1 h, 4 h, and 8 h after injection. Images were captured by a Zeiss LSM780 inverted confocal microscope. Scale bars = 150 µm.

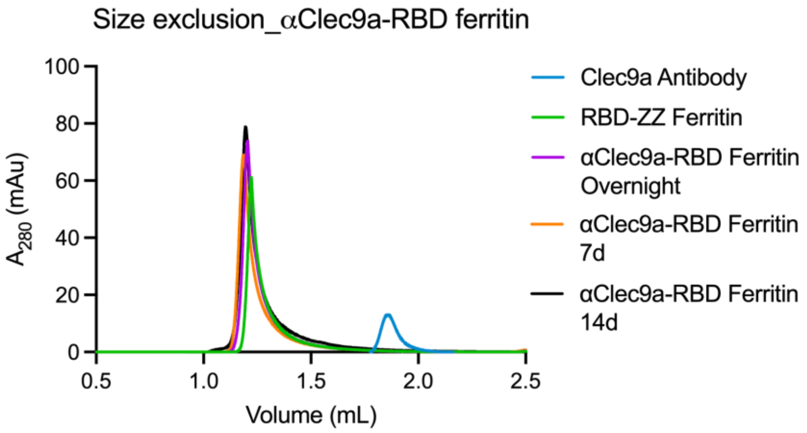

Figure S6: Size exclusion fractions showing the association and stability of Clec9a antibody binding to RBD-ZZ ferritin nanoparticles after overnight incubation and 7 days and 14 days in storage conditions 4 °C.

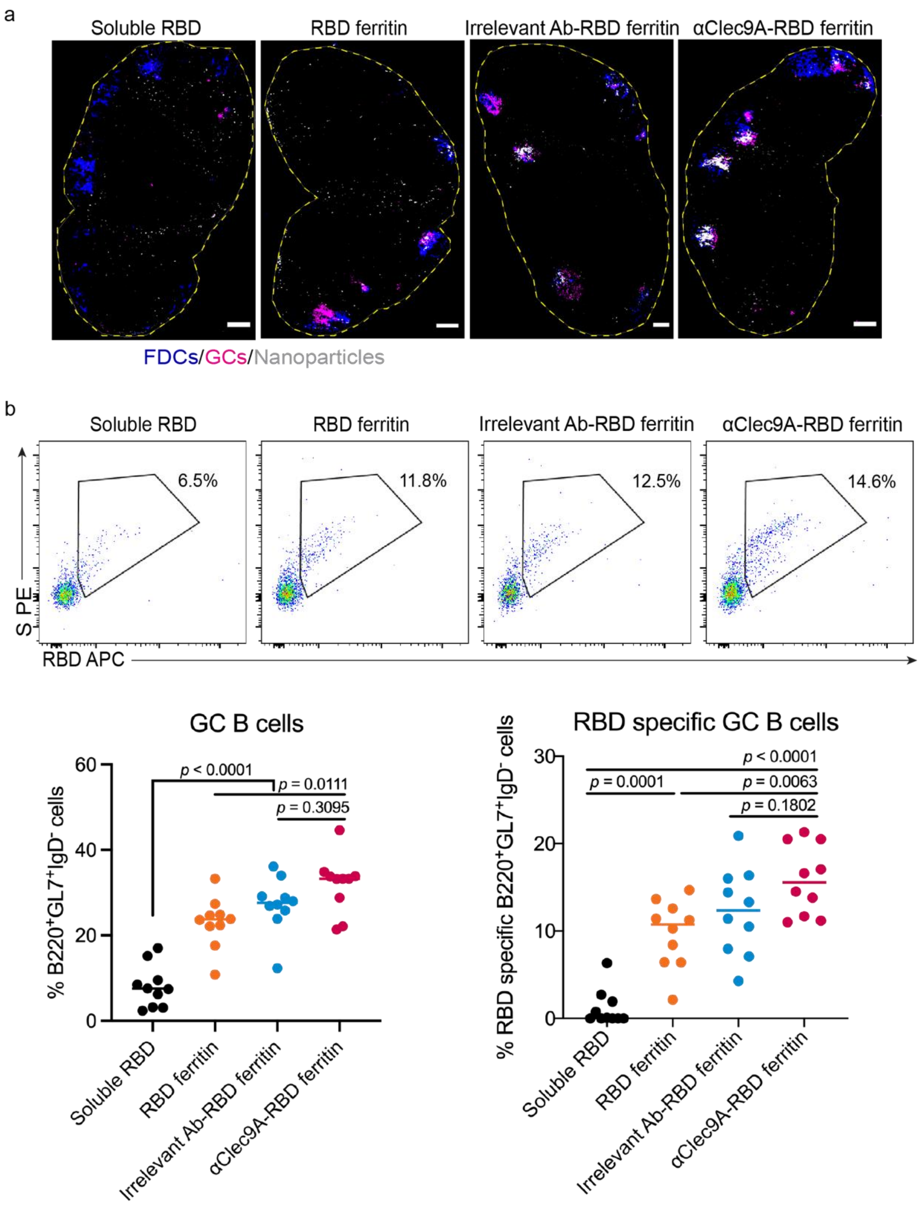

Figure S7: Germinal centre responses in lymph nodes of mice immunised with αClec9a-RBD ferritin. Four groups of C57BL/6 mice (n = 5) were intramuscularly immunised with either soluble RBD, mixture of plain ferritin and RBD-ferritin, irrelevant antibody-RBD ferritin, or αClec9a-OVA ferritin at an equivalent amount of 4.2 µg RBD antigen and 3.0 µg ferritin. At day 21 post prime, inguinal and iliac lymph nodes were harvested and processed. **a**, Deposition of vaccines in FDC and GC areas of draining lymph nodes. Distribution of vaccines fluorescently labelled with AF647 (gray) in lymph nodes (in yellow boundaries) stained with CD35 BV421 (FDC– blue) and GL7 AF488 (GCs – magenta). Images were captured by a Zeiss LSM780 inverted confocal microscope. Scale bars = 150 µm. **b**, Representative flow plot of RBD-specific GC B cell populations ($IgD^{-}B220^{+}GL7^{+}CD38^{lo}$) (top) and frequencies of GC B cells in total $B220^{+}IgD^{-}$ B cells (bottom, left) and RBD-specific GC B cells in total GC B cells (bottom, right). Each dot represents one mouse and data are from two independent experiments. Statistical significance was determined by one-way ANOVA with Tukey's pairwise comparisons post-hoc test.
